# The use of xenografts with fish skin for the treatment of burns: a systematic review and meta-analysis

**DOI:** 10.1590/acb411326

**Published:** 2026-04-17

**Authors:** Caio Placido Costa Arcanjo, Francisco Placido Nogueira Arcanjo, Luiz Odorico Monteiro de Andrade, Ivana Cristina de Holanda Cunha Barreto

**Affiliations:** 1 Universidade Federal do Ceará – Programa de Pós-Graduação em Ciências da Saúde – Sobral (CE) – Brazil.; 2 Fundação Oswaldo Cruz – Eusébio (CE) – Brazil.

**Keywords:** Burns, Skin, Fishes, Tilapia, Systematic Review, Meta-Analysis

## Abstract

**Purpose:**

To conduct a systematic literature review and meta-analysis to assess the efficacy of fish skin xenografts in burn treatment, comparing reepithelialization time, pain reduction, and the amount of analgesics used.

**Methods:**

This review was designed and conducted according to Preferred Reporting Items for Systematic Reviews and Meta-Analysis (PRISMA) guidelines and registered in PROSPERO under registration number CRD42023412250. The databases MEDLINE (via PubMed), Cochrane Library, Scientific Electronic Library Online (SciELO)/Latin American and Caribbean Health Sciences Literature (LILACS), and EMBASE were used. The literature search identified 768 articles. Through title and abstract screening, 736 were excluded, along with 16 duplicates. Sixteen articles were fully analyzed, of which six met the inclusion criteria for this review.

**Results:**

The systematic review found that patients treated with fish skin xenografts had shorter reepithelialization times and reduced pain compared to conventional treatments. The meta-analysis showed reduction in reepithelialization time for the intervention groups using fish skin grafts by 1.26 day (95% confidence interval [95%CI] -2.38 to -0.15; *p* < 0.00001) and a reduction in pain levels with score decreases of 3.84 (95%CI -6.42 to -1.26; *p* = 0.0009). The objective of assessing the number of analgesics used could not be evaluated quantitatively.

**Conclusion:**

The use of fish skin xenografts in burn patients is effective in reducing reepithelialization time and decreasing pain levels during treatment.

## Introduction

According to the World Health Organization, fire burns are responsible for approximately 180,000 deaths annually, making them a global public health issue^1^. Over 95% of fatal fire-related burns occur in low- and middle-income countries, and non-fatal burns are a leading cause of morbidity, prolonged hospitalization, disfigurement, and disability, often resulting in stigma and social rejection. Additionally, burns are among the leading causes of disability-adjusted life years lost in these countries^2,3^. The healing process of burn wounds is complex and dynamic, beginning immediately after the injury and continuing for months or even years as the scar undergoes remodeling^2,4^.

As second-degree burns become deeper, fewer skin appendages remain, meaning deeper burns require more time to heal. Any wound that takes longer than two or three weeks to reepithelialize has a high likelihood of developing into a hypertrophic scar. Therefore, generally, any wound that takes longer than two or three weeks to heal should be considered for excision and skin grafting to reduce the chances of hypertrophic scarring. The goal of treating partial-thickness wounds is to promote reepithelialization^5-7^.

Early excision and skin grafting for burns performed between the second and the 12t^h^ day post-burn is the most important procedure during the patient’s hospital stay, as it is directly associated with improved survival rates. Two types of grafts can be used to cover the wound bed—skin replacement and skin substitute—, which help prevent the entry of microorganisms, prevent dehydration of the area, and accelerate the healing process^8^.

One disadvantage of skin grafts is that the larger the burned area, the more healthy skin must be harvested from the patient. If the burned area is too large, there will not be enough healthy skin to create a complete graft. In such cases, a xenograft is an alternative option for temporary wound coverage. Products derived from pigs are commonly used, but they pose a risk of invasive infections if left in place for more than a few days^8^. Recently, researchers in Brazil have been experimenting with a new treatment for severe burns: the use of fish skin xenografts, an unconventional procedure that reduces the suffering of patients with extensive burns^9^.

For use in the treatment of second- and third-degree burns, tilapia skin has emerged as an economical and effective option. Its moisture content and high levels of type I collagen, comparable to human skin, help heal while promoting recovery and preventing wounds^10^.

In this way, the use of tilapia skin to help heal burn victims is gaining popularity. The skin of this farm-raised fish is an effective and low-cost treatment option due to its abundance, making it a viable alternative to more traditional methods. The skin can be applied directly to the burn site like a bandage, helping to relieve pain and reduce healing time by several days^9^. Interventions using fish skin xenografts for burn treatment have already been carried out, but the evidence regarding the efficacy of this treatment remains unclear.

This systematic review and meta-analysis critically evaluated the current evidence on burn treatment, comparing reepithelialization time, pain levels, and the number of analgesics used between groups treated with fish skin xenografts and those receiving conventional treatment.

## Methods

This is a systematic review and meta-analysis that evaluated the efficacy of fish skin xenografts for burn treatment in reducing treatment time, reported pain, and the number of analgesics used. The study followed the protocol proposed by the Preferred Reporting Items for Systematic Reviews and Meta-Analysis (PRISMA) and was registered in PROSPERO under registration number CRD42023412250. For this study, the databases MEDLINE (via PubMed), Cochrane Library, Scientific Electronic Library Online (SciELO)/Latin American and Caribbean Health Sciences Literature (LILACS), and EMBASE were used, with no restrictions on date or language.

The research question was formulated based on the PICOS description—P: Patients with burns, I: Use of fish skin xenografts for burn coverage, C: Other burn treatment methods, O: Treatment efficacy (recovery time, use of analgesics, pain levels), and S: Clinical trials.

Both randomized and non-randomized clinical studies that used fish skin xenografts for the treatment of burns were included. There were no restrictions on burn type or age group. Studies that did not provide quantitative data were excluded.

To retrieve the articles, the Boolean operators OR and AND were used. For the SciELO/LILACS database, the search algorithms used were (Burns) AND (Tilapia) OR (Fish). In the Cochrane Library, the search algorithms were (Burns), (Tilapia), (Fish), and (Clinical trial) entered without modification. However, to conduct the search in the PubMed/MEDLINE and EMBASE databases, Medical Subject Headings (MeSH) and EMTREE terms were used, respectively. When entering the studied condition (Burns), the platform generated the following terms: (Burns), (Eye Burns), (Burns, Inhalation), (Burns, Electric), (Burns, Chemical). To conduct the search, the term (Burns, Inhalation) was excluded due to its internal nature, as well as (Eye Burns).

A clinical trial filter^11^ was used in combination with the MeSH terms already described: (randomized controlled trial[pt] OR controlled clinical trial[pt] OR randomized controlled trials[mh] OR random allocation[mh] OR double-blind method[mh] OR single-blind method[mh] OR clinical trial[pt] OR clinical trials[mh] OR (“clinical trial”[tw]) OR ((singl*[tw] OR doubl*[tw] OR trebl*[tw] OR tripl*[tw]) AND (mask*[tw] OR blind*[tw])) OR (“latinsquare”[tw]) OR placebos[mh] OR placebo*[tw] OR random*[tw] OR research design[mh] OR follow-up studies[mh] OR prospective studies[mh] OR cross-over studies[mh] OR control*[tw] OR prospectiv*[tw] OR volunteer*[tw]) NOT (animal[mh] NOT human[mh]).

To search the EMBASE platform, in addition to the terms listed above, the EMTREE terms were used: ‘burns’/exp AND ‘tilapia’/exp AND ‘fish’/exp. In addition to this search strategy, a manual search was conducted on the references cited by the selected articles that were potentially eligible and not covered by the previous database searches.

The extracted data were evaluated by two reviewers.

To ensure comprehensive coverage of the literature, we reviewed the reference lists of the included studies and relevant reviews identified through manual searches. After identifying the articles, we exported them to EndNote basic (©2015 THOMSON REUTERS) to remove potential duplicates.

The subsequent phase involved a full analysis of the studies included in the previous phase. This process ensured the integrity and reliability of our systematic review.

A flow diagram was created following the PRISMA model, which includes the stages of Identification, Screening, Eligibility, and Inclusion of items, along with quantities. Using the EndNote program, duplicate articles were independently removed. To assess the risk of bias in the included studies, we employed the Risk of Bias 2.0 (ROB 2.0) software. This tool evaluates bias risk through five domains:

- Bias arising from the randomization process: used to generate the random allocation sequence of participants, which must be random;
- Bias due to deviations from intended interventions: this domain concerns whether the patient and study team were blinded to the group assignment and whether deviations from the proposed intervention occurred that could affect the outcome;
- Bias due to missing outcome data: study participant follow-up loss and, if any, the reason for its occurrence;
- Bias in outcome measurement: assessment of the outcome variable, with the participant, researcher, or data collector unaware of the group assignments;
- Bias in the selection of reported results: the possibility that researchers assess results through multiple evaluations but report only the most convenient ones.

**Figure 1:**
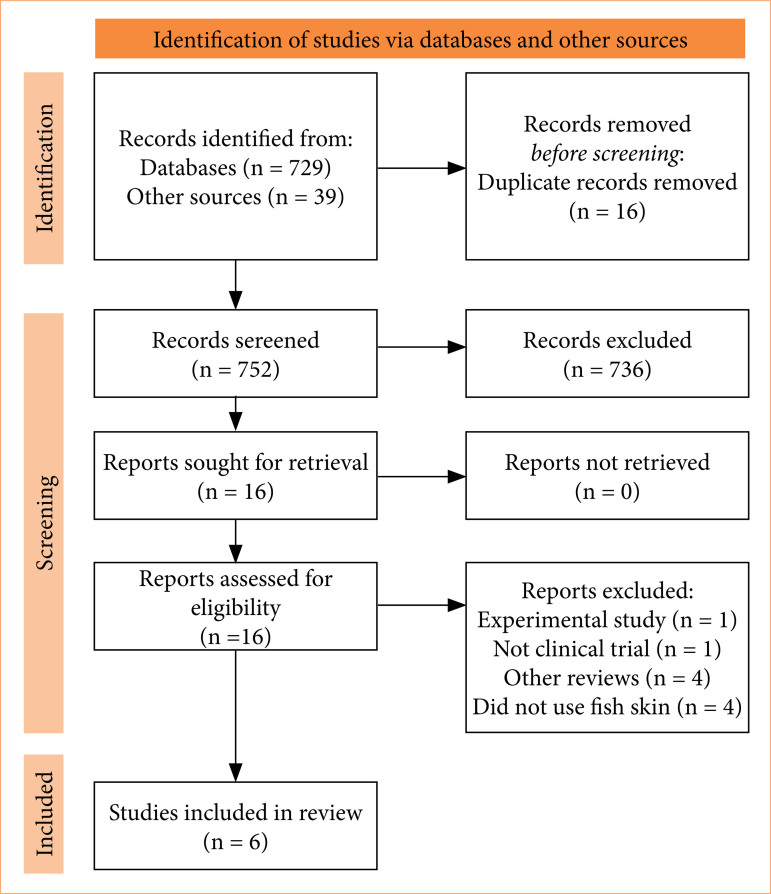
Flowchart of articles searched for the systematic review and selection criteria^12^.

Source: Elaborated by the authors.

The literature search identified 768 articles in the databases, with 39 found through other sources. Sixteen duplicate articles were excluded, leaving 752 articles for the next phase. After applying exclusion criteria, based on titles and abstracts, 16 articles were selected for full-text analysis (Fig. 1).

## Results

After full-text analysis, articles that did not demonstrate applicable quantitative data on the proposed outcomes in this review were excluded. Table 1 summarizes the recovery time for burns, the reported pain levels, and the analgesics used in the studies that were selected^13-18^.

**Table 1:** Description of the studies by analyzed outcome after applying inclusion criteria.

Authors (year)	Sample	Outcome		
		Recovery time (in days)	Analgesics	Pain level
Miranda and Brandt (2019) ^13^	30 participants (15 in each group)	IG: 9.6 ± 2.4 × CG: 10.7 ± 4.5	No data	66.7٪ of those treated with Nile tilapia skin reported a decrease in painful events
Lima Júnior et al. (2020)^14^	30 children (ages 2 to 12), 15 per group	IG: 10.07 ± 0.46 × CG: 0.47 ± 0.74	Dipyrone and/or tramadol	IG: 21.7 ± 8.5 × CG: 27.47 ± 10.0
Lima Júnior et al. (2021)^15^	115 participants (IG = 57; CG = 58)	IG: 9.7 ± 0.6 × CG: 10.2 ± 0.9	Tramadol / ketamine intravenously	IG: 20.5 ± 8.4 × CG: 29.2 ± 13.1
Lima Júnior et al. (2020)^16^	62 participants (IG = 32; CG = 30)	IG: 12.59 ± 3.89 × CG: 14.73 ± 4.82	Dipyrone and/or tramadol	IG: 7.67 ± 2.59 × CG: 8.6 ± 1.9
Lima Júnior et al. (2021)^17^	24 participants (12 in each group)	IG: 9.6 ± 2.4 × GC: 10.7 ± 4.5	Tramadol / ketamine intravenously	IG: 10.21 ± 12.54 × CG: 13.96 ± 8.76
Wallner et al. (2022) ^18^	27 participants (IG = 8; CG = 15)	IG: 22.0 ± 6.3 × CG: 40.5 ± 10.97	No data	IG: 2.8 ± 0.3 × CG: 6.13 ± 2.75

IG: intervention group; CG: control group. Source: Elaborated by the authors.

**Figure 2:**
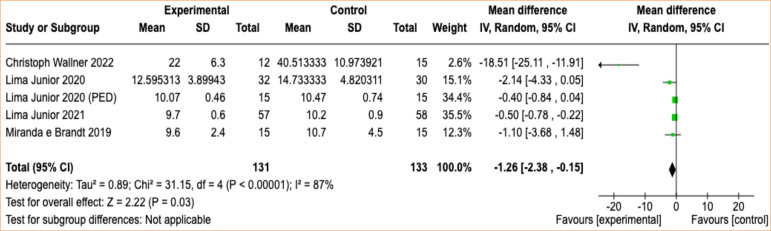
Reduction in recovery time for the intervention groups with fish skin grafts compared to the group receiving conventional treatments.

**Figure 3:**
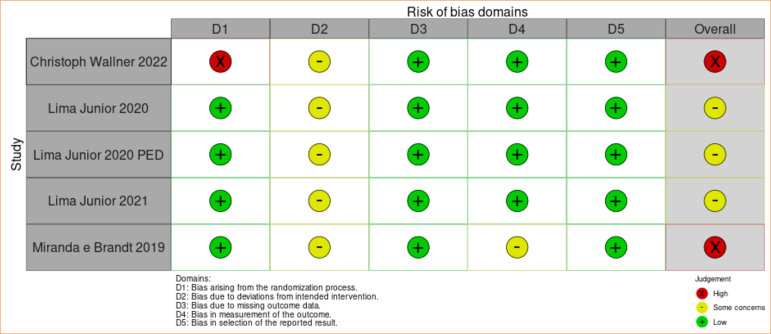
Assessment of the risk of bias for the outcome of reduction in reepithelialization time applied through ROB 2.0.

Source: Elaborated by the authors.

The reduction in recovery time for the intervention groups with fish skin grafts was 1.26 day (95% confidence interval [95%CI] -2.38 to -0.15; *p* < 0.00001; I^2^ = 87%) compared to the group receiving conventional treatments (Figs. 2 and 3).

95%CI: 95% confidence interval. Source: Elaborated by the authors.

**Figure 4:**
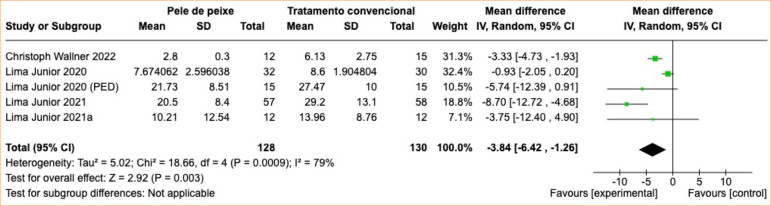
Reduction in pain level for the intervention groups with fish skin grafts compared to the group receiving conventional treatments.

Source: Elaborated by the authors.

**Figure 5:**
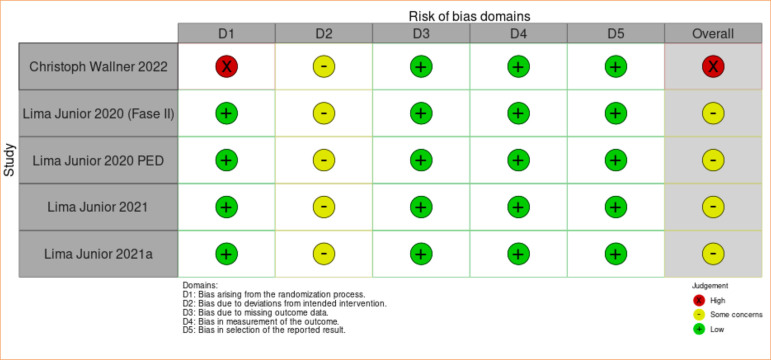
Assessment of the risk of bias for the outcome of reduction in pain level through ROB 2.0.

Source: Elaborated by the authors.

During the analysis of the outcome of pain level, a statistically significant decrease in pain level was observed, with an average reduction in scores of 3.84 (95%CI -6.42 to -1.26; *p* = 0.0009; I^2^ = 79%). Patients in the intervention group experienced less pain, quantitatively represented on the Visual Analog Scale (VAS), compared to the control group. Furthermore, patients treated with fish skin reported a reduction in analgesic needs, indicating an improvement in pain control with statistically significant meta-analysis (Figs. 4 and 5).

The analysis of the analgesics and anesthetics used in the treatment of burns identified a wide variety of therapeutic options for managing pain in burn patients, ranging from topical analgesics to opioids and intravenous anesthetics. A representative meta-analysis of the outcome regarding the quantity of analgesics could not be performed due to the lack of quantitative records, absence of standardized dosing, and the significant variety of analgesics used.

## Discussion

Our study objective was to evaluate the reepithelialization time, pain reduction, and quantity of analgesics in patients undergoing fish skin xenografts compared to conventional treatments.

For the outcome of reepithelialization time, five clinical trials were analyzed individually^13-17^. The study conducted by Lima Júnior et al.^16^, involving 62 patients with tilapia skin, showed a statistically significant reduction in treatment time across the three arms of the study, in which each group differed by the percentage of burned area and the depth of skin involvement. The study arm that showed the greatest reduction in healing time was the group that included patients with 5–10% body surface area affected by second-degree full-thickness or deep burns (group A). This reduction was 3.2 days, with a range of 2.05 to 4.35 days. In another study, Lima Júnior et al.^15^, which evaluated 115 patients, also demonstrated a reduction in treatment time in the intervention group (9.7 ± 0.6) compared to the control group (10.2 ± 0.9). The pilot study conducted by Lima Júnior et al.^14^ with 30 pediatric patients aged 2 to 12 years old admitted within 72 hours of the burn with a burned body surface area of less than 20% did not show a statistically significant reduction in recovery time, although it did show a positive difference in the intervention group. Miranda et al.^13^, in a study involving 30 patients, did not show a statistically significant difference either, despite demonstrating a shorter treatment time in the intervention group (9.6 ± 2.4) compared to the control group (10.7 ± 4.5). The lack of statistical significance in the outcomes of the analyzed articles may be related to a smaller sample size in the groups, as well as greater heterogeneity among the evaluated burns (superficial and deep second-degree).

In a study conducted with North Atlantic cod skin by Wallner et al.^18^, involving 27 patients, the intervention compared the use of the xenograft to two other conventional treatments: synthetic graft (Suprathel) and partial skin graft. The reepithelialization time was shorter, averaging 22 (± 6.3) days in the intervention group, 45 (± 6.6) days in the synthetic graft group, and 34.7 (± 12.5) days in the partial skin graft group, showing a significant reduction in reepithelialization time in the group selected for xenografting. A notable aspect of this study was the evaluation, after 12 months, of a set of characteristics related to graft quality, such as flexibility, thickness, vascularization, and pigmentation, demonstrating the superiority of fish skin over synthetic grafts.

In our meta-analysis, statistical superiority was identified for the use of the xenograft with fish skin regarding the outcome of reepithelialization time. According to these findings, the use of the xenograft with fish skin demonstrated a significant reduction of 1.26 day (95%CI -2.38 to -0.15; *p* < 0.00001) compared to conventional treatment. The comparison was objective due to the use of strictly quantitative data, but it was limited by the variation in burn depth and the wide age range of the evaluated patients.

To assess the reduction in pain levels, the analyzed studies used the VAS; one of them also used the Von Frey digital algometer^16^, and the study with the pediatric population utilized the Revised Faces Pain Scale converted to a numerical scale^14^.

In the study conducted by Lima Júnior et al.^16^, there was a significant improvement in pain levels in the groups subjected to xenografts with tilapia skin, which had a larger burned body surface area and deeper lesions (groups B and C). In contrast, Lima et al.^15^ observed that the use of the xenograft demonstrated non-inferiority in the pain level assessment of the intervention group compared to the control group, similar to the findings of Miranda et al.13, in which no significant difference was noted between the groups (*p* > 0.68). In the study by Lima et al.^17^, the pain level of 24 patients with more than 10% of the body surface burned was analyzed before and after the dressing change, showing a significant reduction in VAS scores for pain after dressing changes in the intervention group (13.96 ± 8.76) compared to the control group (24.79 ± 11.05). In this study, the use of the xenograft reduced pain after dressing changes compared to conventional treatment, along with a lower need for dressing changes, thereby reducing the painful episodes the patient would need to undergo during their treatment.

Wallner et al.^18^ demonstrated a lower pain score in the group treated with North Atlantic cod skin compared to the control group with synthetic grafts, scoring 1.4 (± 0.2) and 3.9 (±0.8), respectively. When comparing the xenograft group and the partial skin graft group, there was no significant variation in pain reports, but there was a parallel analysis that assessed discomfort and itching, also using a VAS, which showed a significant statistical variation favoring the intervention group: xenograft (1.4 ± 0.3), synthetic graft (4.6 ± 0.7), and partial skin graft (2 ± 0.2).

In the only study conducted with a pediatric population14, the VAS was adapted to the Facial Pain Scale and was applied directly to patients older than 5 years old or answered by their caregiver if younger than 5. In the analysis of the outcome, there was no statistical significance in pain reduction, but the reduction in the need for dressing changes in the intervention group (3.0 ± 0.76) compared to the control group (9.27 ± 1.39) was statistically significant. The control group followed a routine of daily dressing changes with silver sulfadiazine, with or without anesthesia, which could improve the child’s well-being and reduce anxiety during hospitalization.

In our meta-analysis, there was a significant reduction in the level of pain in patients in the group treated with fish skin xenografts compared to the control group, with an average pain score reduction of 3.84 (95%CI -6.42 to -1.26; *p* = 0.0009).

In a systematic review conducted by Luze et al.^19^, the use of fish skin xenografts was evaluated, demonstrating accelerated recovery, reduced pain, fewer dressing changes, better cost-effectiveness, and improved aesthetic results compared to conventional treatments for burn victims. This review included case reports, and clinical trials in animals and humans. The results were not quantified, and no meta-analysis was performed for any outcome.

The distinguishing feature of our systematic review is the inclusion, up to the present moment, of all human studies that used fish skin as a xenograft, analyzing quantitative data that statistically demonstrated in a meta-analysis the reduction in treatment time and pain levels in patients undergoing treatment with the xenograft.

Outcomes such as tolerability and the number of dressings showed questionable additional benefits of xenograft therapy, but without quantitative data and standardized analysis that would allow for statistical evaluation.

The patient sample in the groups with data collected in a standardized manner made statistical measures and meta-analysis applicable in two of the three outcomes. In our systematic review, we had 264 patients studied for the outcome of treatment time and 258 patients for the outcome of pain level. The meta-analysis for the third outcome, the quantity of analgesics, proved impossible to conduct due to the lack of quantified data and descriptions in the analyzed articles, as well as the lack of standardization among the medications and doses used in the clinical trials.

Because fish skin is easily visible and therefore distinguishable between the intervention and control groups, there were technical difficulties in blinding the interventions, affecting the analysis through ROB 2.0 in its second domain. Furthermore, the heterogeneity of age, burn degree, and percentage of total body surface area burned among the patients may have influenced the results, being considered limiting factors for a more detailed analysis.

**Figure 6:**
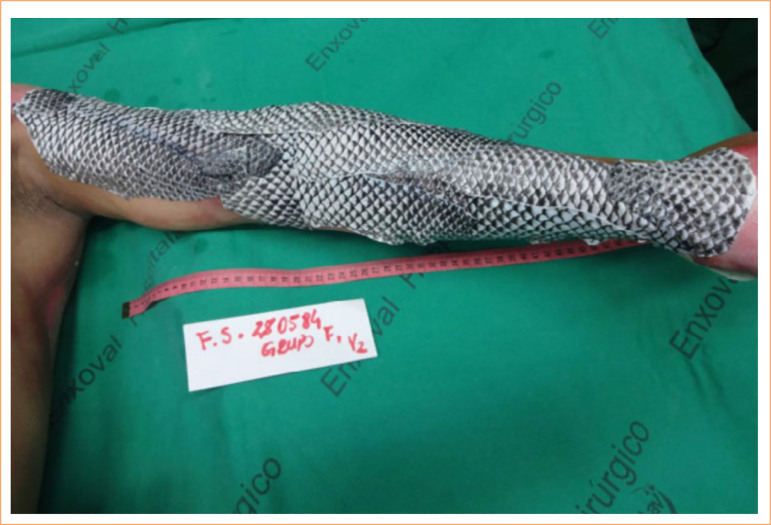
Application of tilapia skin to a partial-thickness burn.

Source: Adapted from Lima-Junior et al.^20^.

**Figure 7:**
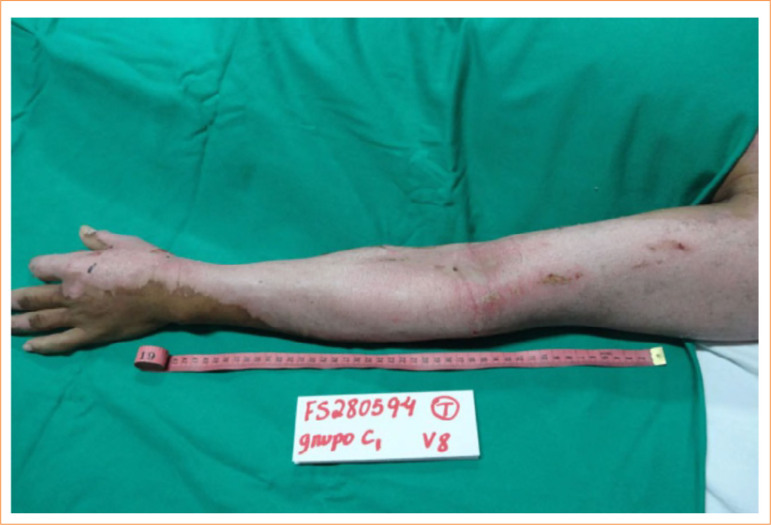
Appearance of the left upper limb wound after the removal of Nile tilapia fish skin showing that complete reepithelialization of the deep partial-thickness burn required a total of 17 days.

Source: Adapted from Lima-Junior et al.^20^.

Despite these methodological limitations, the clinical outcomes observed with Nile tilapia fish skin remain noteworthy. As illustrated in Fig. 6, Nile tilapia fish skin provided immediate and uniform coverage of the affected limb, maintaining a moist environment conducive to tissue repair. Upon removal, the wound demonstrated an organized epithelial layer (Fig. 7), with complete reepithelialization achieved in 17 days, aligning with previously reported results for deep partial-thickness burns treated with Nile tilapia fish skin^20^. These findings reinforce the potential of Nile tilapia fish skin as a biologically active, cost-effective alternative to conventional dressings and suggest that, even in the presence of heterogeneity and challenges in blinding, the intervention may offer meaningful clinical benefits.

## Conclusion

The evidence shows that the use of fish skin xenografts in patients with superficial or deep second-degree burns is effective in reducing the time to reepithelialization and may decrease the level of pain during treatment. The current gold standard is the use of partial skin grafts for treating these burns, but this strategy has limitations, such as the percentage of total body surface area burned. The results of this meta-analysis confirm its effectiveness in reducing treatment time and the potential for improving pain during treatment, making the use of fish skin a promising option for a new, effective, and safe treatment.

## Data Availability

The data will be available upon request.
